# Integrated optimization of urban rail transit line planning, timetabling and rolling stock scheduling

**DOI:** 10.1371/journal.pone.0285932

**Published:** 2023-05-18

**Authors:** Chao Li, Jinjin Tang, Jun Zhang, Qingqing Zhao, Lingli Wang, Jian Li

**Affiliations:** 1 School of Traffic and Transportation, Beijing Jiaotong University, Beijing, China; 2 Second Planning Institute for Comprehensive Transport, Nanjing Institute of City & Transport Planning Company Limited, Nanjing, Jiangsu, China; 3 Beijing Subway Operation Corporation, Beijing, China; University of Hong Kong, HONG KONG

## Abstract

Urban rail transit train operation plan is a comprehensive production plan encompassing line planning, timetabling, and rolling stock scheduling. In order to solve the problem of infeasibility of the line plan and timetable because the number of rolling stocks could be only precisely considered in the rolling stock scheduling. An integrated optimization solution is proposed which considers the line plan, timetable, and rolling stock schedule. Candidate service routes are generated according to the layout of the turn-back stations. Considering the constraints of operation and passenger flow demand, an integer nonlinear programming model is established to minimize the cost of operation and passenger waiting time. The model complexity is analyzed and based on its decomposability a deterministic search algorithm is designed. Taking Chongqing Metro Line 3 in China as an example to verify the effectiveness of the proposed model and algorithm. Compared with the train operation plan based on manual experience and compiled by stages, the integrated optimization model can better improve the quality of train operation plan.

## Introduction

Urban rail transit has become one of the most effective ways to relieve traffic congestion because of its environmental protection, punctuality, and efficient transportation characteristics [1, 2]. The train operation plan of urban rail transit organizes train operation internally and provides transport service externally [3]. Its quality directly affects the efficiency of train transportation organization. In the process of train operation planning, operation planners usually consider both the operating enterprises and passengers [4]. From the perspective of operation enterprises, they are mainly concerned about the safety, efficiency, cost of train operation, and the difficulty of operation organization. From the perspective of passengers, high-quality and convenient transportation services should be provided. For example, the travel time and the load factor of trains should be reduced. Passengers usually hope to make train operation plans with full-length service route and high frequency to improve travel efficiency, but such train operation organization measures would increase the number of trains, which leads to an increase in operation costs. Therefore, how to balance the operating cost and passenger service level is the key to optimizing the train operation plan of urban rail transit.

**Fig 1:**
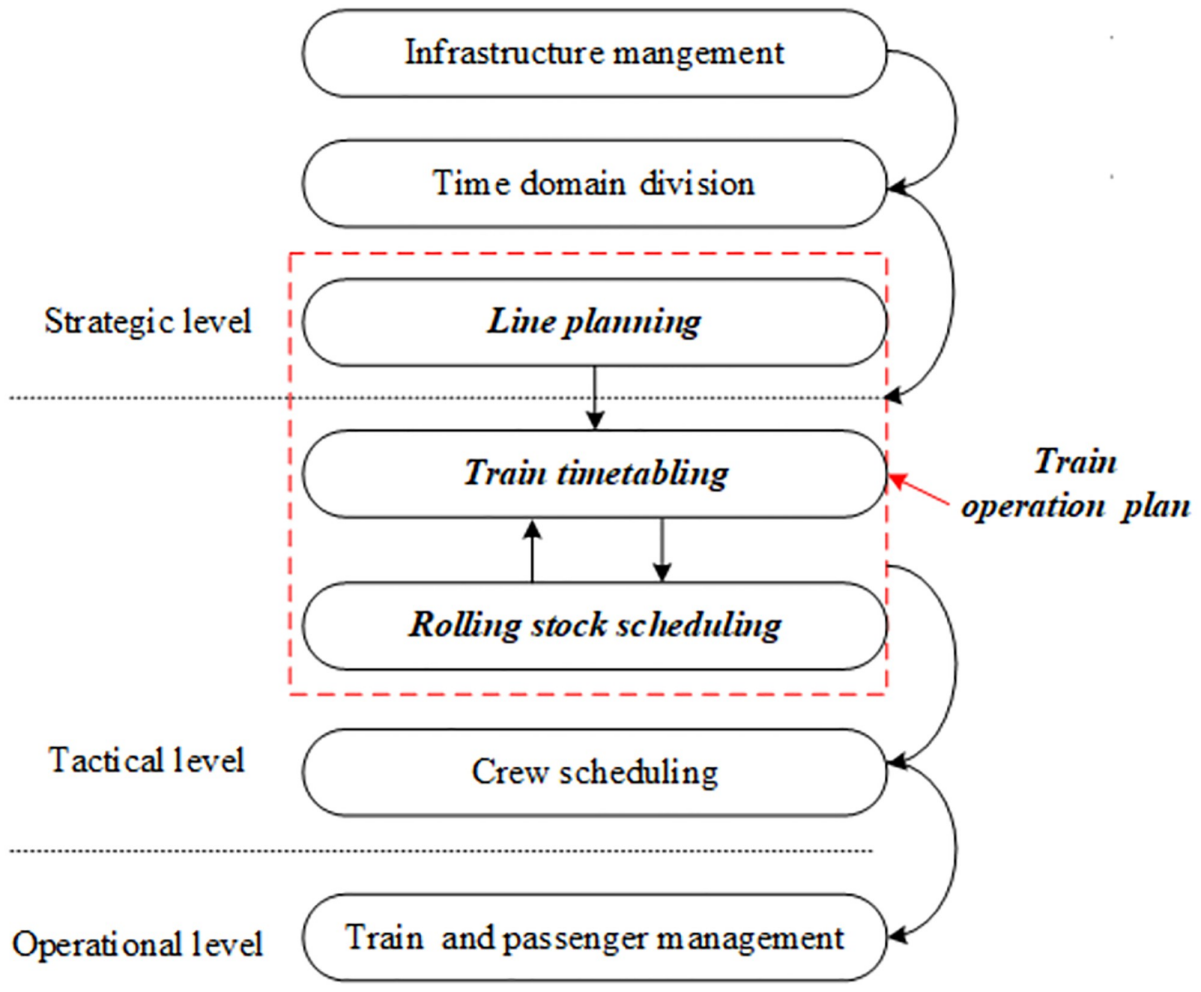
Urban rail transit hierarchical planning process and research scope of this paper.

As a key technical document underpinning the operation of urban rail transit services, train operation plan mainly includes line plan, timetable, and rolling stock schedule [5]. Owing to the inherent complexity of the urban rail traffic system, the urban rail transit planning process shown in Fig 1 is usually hierarchically divided into several stages to reduce computational complexity. Line planning is a key operational problem which must be solved by metro operating company from the strategic level. Based on the capacity of line fixed facilities and equipment and passenger flow demand, it is a problem to determine train service routes, train composition, and frequency of each service route during the study period. Then, taking the output of the line planning problem as the input, the timetabling problem focuses on optimizing the arrival and departure time of each train at the station on the railway line. Finally, based on the arrival and departure time of trains, the rolling stock scheduling determines the circulation sequence and time of trains at the turn-back stations. Significantly, timetabling and rolling stock scheduling affect each other. The timetable can be adjusted by altering the circulation sequence and time of trains at the turn-back stations.

The train operation planning problem is divided into the above three subproblems for optimization to improve the solving efficiency [6]. These three subproblems are correlated, and the solution of one subproblem will limit the optimality of the next subproblem in the process of stepwise optimization. In addition, the calculation of passenger travel cost and operating cost often relies on the solution of multiple subproblems, rather than a single subproblem. Taking passenger waiting time as an example, it depends not only on the train running frequency obtained in the line planning subproblem, but also on the arrival and departure time of each train obtained by solving the timetabling subproblem. Similarly, the operating cost of an enterprise is not only related to the train service route determined by the line planning subproblem, but also related to the number of rolling stocks determined by the rolling stock scheduling subproblem. In a word, it is necessary to make the three subproblems of line planning, timetabling, and rolling stock scheduling as an inter-connected whole, and achieve their integrated optimization.

More and more scholars have noticed the importance of integrated optimization of all stages of train operation plan. Yan and Goverde [7] studied line planning and timetabling integration optimization of high-speed railway. Wang et al. [8] discussed the relationship between frequency of train service routes and train scheduling. Blanco further studies the integrated optimization of line planning and timetabling with short-turn service routes. Schöbel [5] proposed an eigenmodel of route planning, timetabling, and rolling stock scheduling for public transportation. Zhou and Mehdi [6] extended the generalized model to urban rail transit, but it only considered the operation organization of the full-length service route and the special situation of a single depot layout. Although the existing studies provide a good reference for the integrated optimization of urban rail transit train operation plan, there are few studies on integrated optimization considering line plan, train timetable, and rolling stock schedule. With the extension of the line, the multiple service routes and multiple train compositions gradually become the main train operation organization mode in the actual operation process. To fill the gap between theoretical research and practical application, this study also needs to further consider the actual operation situation of multiple routes and multiple train compositions, the restriction of the fleet size used on the train operation plan, and the limitation of the train’s turn-back running at the turn-back station. The research focus of this paper is as follows:

- This paper takes the train operation plan during the morning peak period as the research object and generates the candidate service routes according to the layout of the turn-back station on the line. To fill the gap in the integrated optimization of line planning, train timetable, and rolling stock schedule, concepts of the running offset of opposite trains and train running standard station are introduced to realize the coupling among line planning variables, train timetable variables, and rolling stock schedule variables.
- Based on the capacity of line fixed facilities and fleet size, this paper establishes a general integer nonlinear programming model for the integration optimization of train operation plan, aiming at minimizing the operation cost and passenger waiting time cost. The model is general since it considers the operation organization strategy of multiple service routes and multiple train compositions.
- A tailored and easy to implement deterministic search algorithm is developed to solve instances efficiently. It starts from the easily available feasible solution, and iteratively solves the three sub-models in a certain search range with a fixed step size. With a reasonable parameter setting, the algorithm can be used to find optimal or near-optimal solutions for instances within acceptable computation time.
- This research demonstrates the effectiveness of the integrated model by comparing the optimization results of the model with the train operation plan compiled based on manual experience in terms of operating cost and passenger service level. In addition, the sensitivity analysis is carried out for the fleet size, the maximum number of service routes operated by the line, and the weight coefficient of the objective function, which proves the practicability and reliability of the integrated model.

The remainder of this paper is organized as follows. First, a brief literature review is presented. Second, the problem is described. Third, the integrated optimization model of line planning, timetabling, and rolling stock scheduling is formulated. Fourth, a deterministic search algorithm is developed. Fifth, based on case studies on a real-world metro line, the effectiveness of the proposed model is discussed. Finally, the main conclusions are summarized.

## Literature review

In a sense, the train service design of an urban rail transit line is like the line planning of high-speed railways. Based on the capacity of line equipment, both take passenger flow as input to determine the train routes and frequencies [9–11]. Sun et al. [12] constructed a frequency optimization model aiming at maximizing the train load rate while minimizing the number of trains. Niu et al. [13] built an optimization model of unbalanced train operation scheme in crowded environment. Taking passengers’ choice into consideration, Li et al. [14] established a model of line planning for Y-type line. However, the above studies did not consider the unbalanced distribution of passenger flow in each station of the line. To solve this problem, Deng et al. [15] built an urban rail transit line plan optimization model aiming for the minimum generalized travel cost of passengers, the lowest operating cost of enterprises, and the least number of operating hours, and short-turn service routes are considered in the model. Xu et al. [16] built an optimization model of line planning based on passenger flow demand which considered short-turn routing mode. To further improve the level of passenger service, the train operation organization including multiple train compositions has been paid more and more attention. Li et al. [4] built a train service design model with multiple routes and multiple train compositions under capacity constraint.

In general, the problem of timetabling can be divided into cyclic and non-cyclic. Most studies on the cyclic train timetabling problem are based on periodic event scheduling problem (PESP) modeling framework. Liebchen [17], optimized the cyclic train schedule of the Berlin Metro to minimize the waiting time for passenger transfers. Zhang et al. [18] constructed an integer linear programming model for the cyclic train timetabling problem through the space-time network and used the ADMM algorithm to solve it. Based on the PESP model, numerous new models have been developed for practical cyclic train timetabling problems, including capacity evaluation [19] and robust train timetables [20]. For the non-cyclic train timetabling problem, Higgins et al. [21] used the big M method to model the traffic conflict between trains. Zhou et al. [22] aimed to minimize the cost of train operations by constructing a space-time-speed network to optimize the non-cyclic train timetable. For the hybrid cyclic train timetable, Robenek et al. [23] combined the regularity of the cyclic timetable with the flexibility of the non-cyclic timetable to improve passenger satisfaction. Yin et al. [24] constructed a mixed integer programming model based on the periodic event-activity network model to generate a mixed train timetable that meets passenger travel demand.

As the final stage in the compilation of a train operation plan, the rolling stock schedule is mainly addressed from the perspective of the operating company whereby, under the constraint of the fleet size, the rolling stock needs to be positioned in such a way as to meet the timetable scheduling service [25–27]. Peeters et al. [28] constructed a mixed integer linear programming model to determine the allocation of vehicles and train operation plan, and used a branch and bound algorithm to solve the problem. Cadarso and Marín [29] established a model for the robust rolling stock by considering train composition, train empty movements, and passenger flow demand. Mahadikar et al. [30] constructed a multi-stage allocation model with the main objective of minimizing the empty movement mileage of trains by considering the capacity of the depot and the demand for the number of vehicles on the train service route. Ucar et al. [31] considered two types of disruptions in the scheduling process of the multi-depot vehicle and proposed a unique recovery method to deal with these potential disruptions, and designed a simultaneous column-and-row generation algorithm to find a valid lower bound of the model.

The integrated optimization of each stage in the train operation plan has also attracted the attention of scholars. Aiming at the integrated optimization of line plan and timetable, Kaspi and Raviv [32] sought to integrate the optimization of running line plan and timetable to construct a model to minimize passenger travel time and operating cost. The model was solved using a cross-entropy metaheuristic. Zhang et al. [33] constructed a unified integer linear programming model that coupled the variables of line planning and the variables of train timetabling through a cross-resolution consistency constraint. In terms of the integrated optimization of train timetable and rolling stock schedule, Teng et al. [34] optimized the relation between trains’ departure times so as to smooth the vehicle departure intervals and minimize total charging costs. Carosi et al. [35] constructed a multi-commodity flow model and designed a mathematical heuristic algorithm to solve the integrated model of timetable and rolling stock schedule. Shang et al. [36] constructed an integrated model coupling the space-time network of passenger travel and train running to minimize passenger travel time. Ibarra-Rojas et al. [37] proposed a bi-objective model to deliver collaborative optimization between the rolling stock schedule and train timetable and thus minimize operating costs and passenger travel costs. Michaelis and Schöbel [38] rearranged all stages of the train operation plan to solve the shortcoming of the traditional method, where the fleet size is only considered in the final rolling stock schedule stage.

For comparative convenience, the detailed characteristics of some closely related references are listed in Table 1. Research gaps in existing studies are summarized as follows. Firstly, most of the former works only consider one or two stages of train operation planning. This makes it difficult for the optimization results of the model to guide train running as part of the actual train operation plan. Secondly, in view of the existing line planning, timetabling, and rolling stock scheduling integration optimization research (e.g., Zhou and Mehdi [6], Michaelis and Schöbel [38]), they follow a hypothesis that only the full-length service route exists. When additional service routes are considered, models become intractable. The train operation organization strategy of multiple routes is common and cannot be ignored in the actual operation process of urban rail transit. Finally, existing research especially in the field of rail systems mainly designs service routes whose train composition is pre-determined. Train composition is an important parameter that affects the train capacity and operating cost. The flexible setting of train composition can get a better train operation plan.

**Table 1:** Systematic comparison of related studies.

Publication	Optimization factor(s)	Model type	Decision variables	Objective	Optimal Solution
Fu et al.(2015)	LP	bi-level programming model	MR+SF	Minimize passenger travel time and company costs and maximize the number of passengers on the train	No
Xu et al.(2017)	LP	NLP	MR+SF	Minimize passenger travel cost and operator costs	No
Li et al.(2021)	LP	MINLP	MR+SF+TC+TS	Minimize operator costs and passenger waiting time	Yes
Zhang et al.(2019)	TT	NLP	MR+SF+DT	Minimize train travel costs	No
Zhou et al.(2017)	TT	INLP	MR+TS+DT	Minimize train energy consumption	No
Yin et al.(2019)	TT	MINLP	SR+DT+CS	Minimize train travel time and passenger waiting time	No
Cadarso and Marín et al. (2011)	RS	MILP	CS	Minimize operating costs	Yes
Mahadikar et al. (2015)	RS	MILP	CS	Minimize dead kilometers	Yes
Ucar et al.(2017)	RS	ILP	CS	Minimize operator costs	No
Shang et al.(2021)	TT+RS	SR+DT+CS	ILP	Minimize passenger travel time	No
Zhang et al.(2022)	LP+ TT	ILP	SF+DT	Minimize operator costs and passenger waiting time	No
Schöbel.(2017)	LP+TT+RS	—	—	Minimize passenger travel time and company costs	No
Zhou and Mehdi.(2021)	LP+TT+RS	MINLP	SR+DT+CS	Minimize passengers’ travel costs and enterprise’s operating costs	No
Our paper	LP+TT+RS	NLP	MR+TC+TS+DT+CS	Minimize operator costs and passenger waiting cost	Yes

**Model type**:LP—line planning; TT—timetable; RS—rolling stock schedule; ILP—integer linear programming; MINLP—mixed integer nonlinear programming; INLP—integer nonlinear programming; MILP—mixed integer linear programming; NLP—nonlinear programming;**Decision variables**:SR—single route; MR—multiple routes; TC—train compositions; SF—service frequency; TS—train size; DT—departure time; CS—circulation sequence; “#x2014;”—the corresponding item is not considered.

## Description of the problem

This paper studies the integrated optimization of line planning, timetabling, and rolling stock scheduling within a specified study period in an urban rail transit line. There are two main tracks on the line, each of which is only for the trains running in one direction. To distinguish the directions, trains that run on the upward track are referred to as upward trains, and trains that run in the opposite direction from upward trains are referred to as downward trains. The stations on the line are divided into two categories. The first is the turn-back station where turn-back tracks are built to reverse the running direction of trains. The second type is the ordinary station without turn-back tracks, at which the running direction of trains cannot be reversed. A line usually contains multiple turn-back stations, so there are multiple train service routes. The train service route is composed of two turn-back stations which are used to change the direction of downward trains and upward trains respectively. The service route covering the whole line is defined as the full-length service route. The other service routes that only cover partial stations of the line are defined as short-turn service routes. As the service object of urban rail transit, passenger flow is the main basis for the preparation of train operation plans.

The line plan determines the train service routes, train composition, and frequency of each service route during the study period. Its compilation is mainly affected by passenger flow characteristics and operational rules. The larger the inbound volume, the more trains with large composition will be operated. Short-turn trains usually are operated between turn-back stations with dense passenger flow distribution. The more unbalanced the distribution of passenger flow on the line, the more trains will be operated on short-turn service routes. The train timetable determines the time of arrival and departure at the station for trains that are operated in the line plan. It affects the waiting time of passengers and the rolling stock schedule. Based on timetable, the rolling stock schedule determines the circulation sequence and time of trains at the turn-back stations. Considering the generality of the layout location of the depot, the up and down trains run in pairs during the study period. At the same time, to correlate the departure times of trains with multiple routes, this paper takes the starting station and the ending station in the upward direction of the line as the standard stations for the up and down trains, respectively.

**Fig 2:**
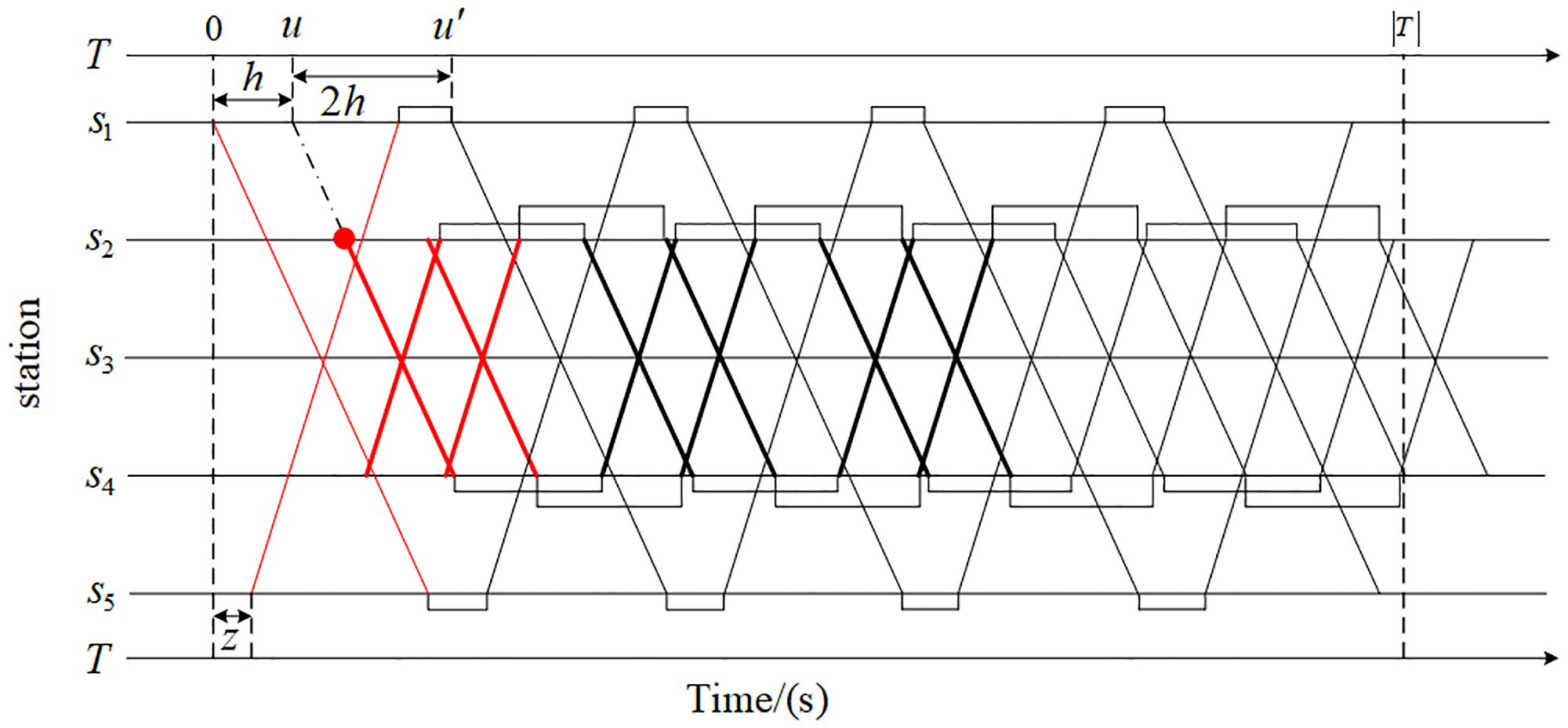
Train operation plan.

To describe the problem more fully, a simple train operation plan has been adopted in this research, as shown in Fig 2. This line consists of five stations and four sections. The stations are denoted as *s*_1_ to *s*_5_ in the downward direction, of which *s*_1_, *s*_2_, *s*_4_ and *s*_5_ are turn-back stations. *s*_1_-*s*_5_ is the full-length service route. *s*_2_-*s*_4_ is a short-turn service route. The maximum allowable train composition for *s*_1_-*s*_5_ is 6-car, in which the number of cars is 6. The maximum allowable train composition for *s*_2_-*s*_4_ is 8-car, in which the number of cars is 8. According to the spatial and temporal distribution characteristics of passenger flow, the line plan, timetable, and rolling stock schedule are determined. As shown in Fig 2, the *s*_1_-*s*_5_ operates 5 pairs of 6-car trains. *s*_2_-*s*_4_ operates 6 pairs of 8-car trains (thick lines) and 4 pairs of 6-car trains. *s*_1_ and *s*_5_ are the standard stations consisting of the upward and downward trains. *h* is the train running interval in the study period. *u* stands for the departure time of the downward train running on the route *s*_2_-*s*_4_ at the standard station *s*_1_, which is used to calculate the departure time *u*′ of the subsequent train. *z* is the offset time of the departure time of the upward train, which is used to couple the departure time of the trains in different running directions.

The number of trains starting from the turn-back station without cycle is the number of rolling stocks required to implement the train operation plan. It is the key to judge whether the train operation plan is feasible. If the number of rolling stocks required for the implementation of the train operation plan exceeds the fleet size, the train operation plan is not implementable. In addition, the number of rolling stocks determines the fixed cost and emergency support capacity of the enterprise. Consequently, reducing the number of rolling stocks used is of great significance to enterprise operation. As for the train operation plan shown in Fig 2, the fourth downward train is taken as the benchmark, and the subsequent downward trains are circulated by upward trains. Therefore, this kind of trains does not need to allocate rolling stock to carry out the transport task. At the same time, three downward trains failed to be circled. Thus, the total number of rolling stocks required in the downward direction is 3. As shown in Fig 2, the total number of rolling stocks required to implement the train operation plan is 6. In addition, the fleet size can be obtained by the number of trains minus the number of train cycles. Taking the train operation plan shown in Fig 2 as an example, the number of trains is 30, and the number of train cycles is 24. Accordingly, the fleet size is 6.

**Fig 3:**
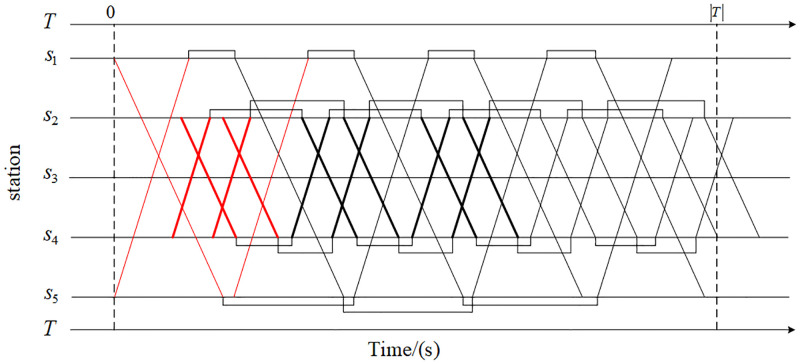
Train operation plan when *z*=0.

The rolling stock schedule determines the circulation sequence and time of trains at the turn-back stations in the train operation plan. The number of rolling stocks required to execute the train operation plan can be accurately calculated only after the rolling stock schedule is determined. Therefore, it is necessary to optimize the rolling stock schedule in train operation planning. Meanwhile, the rolling stock schedule is affected by the timetable. Keep train service routes, train composition, and the number of trains unchanged, adjust the upward train timetable of the train operation plan shown in Fig 2, that is, let *z*=0, and get the train operation plan shown in Fig 3. Considering the constraints of the minimum turn-back time at the turn-back station, the circulation between trains is completed according to the principle of first come, first turn back. The fleet size required for the train operation plan shown in Fig 3 is larger than the train operation plan shown in Fig 2, which further proves the necessity of integrated optimization of the route plan, timetable, and rolling stock schedule.

The train operation plan affects both the operating cost of the enterprise and the service level of the passengers. On the one hand, the train operation plan determines the fleet size and train running cost, thereby determining the operating cost. On the other hand, the different train departure intervals of each station have a significant impact on the waiting time of passengers. Therefore, while formulating the operation plan, besides meeting the basic operating rules and passenger flow requirements, the fleet size should be minimized, running cost of trains should be reduced, and the travel time of passengers should be saved. The line plan, timetable, and rolling stock schedule need to consider both the existing fixed facilities and equipment conditions of the line, as well as the need to meet the passenger flow demand during the period, and to do so in such a way as to minimize the operating cost of the enterprise and the travel cost of passengers. The mentioned above is the integrated optimization problem of an urban rail transit train operation plan.

## Mathematical model

### Model hypothesis

- Each service route operates at least one type of composition train.
- Trains of each service route stop at all stations on the service route.
- Trains have the same running time in each section and the same dwell time at each station of the line.
- In the process of passenger flow allocation, they select the earliest train with the remaining capacity that passes through the passenger’s origin station and destination station.

### Symbol description

Tables 2–4 summarize the main sets, indices, parameters, and variables of the model. Among them, the variable of line planning includes the route selection variable *x*_*r*_ and train running variable *y*_*r*,*f*,*m*_. *y*_*r*,*f*,*m*_ can be used to determine the number of trains running on each route during the study period. Then the frequency of each service route can be determined. Timetabling variables include departure time variables $u_{r,f,m}^{d,s}$, arrival time variables $v_{r,f,m}^{d,s}$. The variable of rolling stock scheduling includes the train cycle variable $\gamma_{r,f,m}^{d,r^{\prime },f^{\prime }}$.

**Table 2:** Definition of sets and indices.

Sets and indices	Definition
*D*	Set of running directions with index *d*, *D* = {1, 2}. 1 is the downward direction, 2 is the upward direction;
*E*	Set of sections with index *e*;
*F*	Set of trains with index *f*. |*F*| is the maximum number of trains that can be operated in a single direction on the line;
*M*	Set of train compositions with index *m*;
*R*	Set of all candidate service routes with index *r*;
*R*_*r*,*d*_	Set of routes in the set *R* that can be used to circulate trains with route *r* and direction *d*;
*S*	Set of stations in the urban rail transit line with indexes *s* and *s*′, *S* = {1, ⋯, |*S*|}. 1 is the downward standard station; |S| is the upward standard station;
*T*	Set of times stamps with index *t*, *T* = {1, ⋯, |*T*|}. 1 is the beginning time of the study period, |*T*| is the end time of the study period;
*P*	Set of passengers with index *p*, *P* = {1, ⋯, |*P*|}. |*P*| is the number of passengers entering the line during the study period;
*P*_*s*,*d*_	Set of passengers traveling in direction *d* and passing through station *s*;
*d*_*s*,*s*′_	Index of direction index from station *s* and station *s*′;
$s_{r,d}^{1}$	Index of the origin station of train running on service route *r* in direction *d*;
$s_{r,d}^{2}$	Index of the destination station of train running on service route *r* in direction *d*;
$s_{p}^{1}$	Index of the origin station of passenger *p*;
$s_{p}^{2}$	Index of the destination station of passenger *p*;
*t*_*p*_	Index of the time passenger *p* arrived at the departure station $s_{p}^{1}$;

**Table 3:** Definition of parameters.

Parameters	Definition
*a*_*e*_	Minimum number of trains that need to run in section *e* within the study period;
*b*_*e*_	Maximum number of trains that need to run in the section *e* within the study period;
$c_{m}^{1}$	Fixed cost of operating a rolling stock with composition *m* within the study period, unit: CNY/rolling stock;
$c_{m}^{2}$	Running cost of operating a train per kilometer with composition *m* within the study period, unit: CNY/ km;
*c*_3_	Passenger’s waiting time cost per hour, unit: CNY/h;
*g*_*r*_	Distance traveled by the train running on service route *r* in single direction, unit: km;
*l*	Expected load of the train, unit: %;
*n*_*m*_	Fleet size of rolling stocks with composition *m*;
*o*_*m*_	Capacity of a train with composition *m*;
$t_{s,s^{\prime }}^{d}$	Sum of the running time and the stopping time of the train running from station *s* to station *s*′ in direction *d*, unit: s;
$t_{s}^{d}$	Stopping time of the train at station *s* in direction *d*, unit: s;
*α*_*r*,*s*_	0–1 parameter, if service route *r* covers station *s*,*α*_*r*,*s*_ is 1, 0 otherwise;
*β*_*r*,*e*_	0–1 parameter, if service route *r* covers section *e*,*β*_*r*,*e*_ is 1, 0 otherwise;
*μ*_*r*,*d*_	Minimum turn-back time of a train in direction *d* on service route *r* at the destination station, unit: s;
*η*	Maximum allowable number of service routes operated on the study line;
*ω*_*e*_	Maximum number of passengers traveling between the upward and downward directions of section *e* within the study period;
λ_*r*_	Maximum number of train compositions allowed to run on service route *r*;
*τ*	Weight of operating costs in the objective function of the model;

**Table 4:** Definition of variables.

Variables	Definition
*h*	intermediate variable, average interval between trains during the study period, unit: s;
$q_{f,m}^{s,d}$	intermediate variable, the number of passengers in the train *f* in direction *d* with composition *m* after leaving station *s*;
$u_{r,f,m}^{d,s}$	intermediate variable, the departure time of the train *f* in direction *d* with composition *m* on service route *r* at station *s*;
$v_{r,f,m}^{d,s}$	intermediate variable, the arrival time of the train *f* in direction *d* with composition *m* on service route at station *s*;
$w_{f,m}^{p}$	0–1 intermediate variable, if passenger *p* is in the boarding time window of the train *f* in direction $d_{s_{p}^{1},s_{p}^{2}}$ with composition *m*, $w_{f,m}^{p}$ is 1, 0 otherwise;
*x*_*r*_	0–1 variable, if service route *r* is adopted by the considered line, *x*_*r*_ is 1, 0 otherwise;
*y*_*r*,*f*,*m*_	0–1 variable, if the train *f* with composition *m* on service route *r* is operated, *y*_*r*,*f*,*m*_ is 1, 0 otherwise;
*z*	Continuous variable, offset of the departure time of the first train in the upward direction at the standard station, unit: s;
$\gamma_{r,f,m}^{d,r^{\prime },f^{\prime }}$	0–1 variable, if the train *f*′ in direction *d* with composition *m* on service route *r*′ circulates the train *f* in direction *d*′ with composition *m* on service route *r*, $\gamma_{r,f,m}^{d,r^{\prime },f^{\prime }}$ is 1, 0 otherwise. Note that *d* and *d*′ are in opposite directions;
$\delta_{r,f,m}^{d,s,t}$	0–1 intermediate variable, if the train *f* in direction *d* with composition *m* on service route *r* doesn’t leave the station *s* at time *t*, $\delta_{r,f,m}^{d,s,t}$ is 1, 0 otherwise;

### Objective function

The operating cost of urban rail transit line mainly includes fixed cost and variable cost. The fixed cost is the total acquisition cost of the rolling stocks used to implement the train operation plan adopted in the line. The variable cost is mainly the total running cost of all the trains put into use on the line during the study period. The fixed cost and operation cost during the study period mainly depend on fleet size and running kilometrage during the study period. The fleet size can be obtained by the number of trains running minus the number of circulations. The fixed cost is shown in Eqs 1 and 2, $\sum_{r\in R}\sum_{f\in F}y_{r,f,m}$ represents the number of trains with composition *m*, 2 represents the number of directions in which trains run, and $\sum_{r\in R}\sum_{f\in F}\sum_{d\in D}\sum_{r^{\prime }\in R_{r,d}}\sum_{f^{\prime }\in F}\gamma_{r,f,m}^{d,r^{\prime },f^{\prime }}$ represents the number of cycles. The variable cost is shown in Eq 2.

$$\begin{array}{c} Z_{11}=\sum_{m\in M}c_{m}^{1}(2\sum_{r\in R}\sum_{f\in F}y_{r,f,m}-\sum_{r\in R}\sum_{f\in F}\sum_{d\in D}\sum_{r^{\prime }\in R_{r,d}}\sum_{f^{\prime }\in F}\gamma_{r,f,m}^{d,r^{\prime },f^{\prime }}) \end{array}$$ (1)

$$\begin{array}{c} Z_{12}=\sum_{r\in R}\sum_{f\in F}\sum_{m\in M}c_{m}^{2}\cdot g_{r}\cdot y_{r,f,m} \end{array}$$ (2)

Passenger travel time is mainly composed of five parts, including access time, origin/transfer waiting time, in-train time, transfer walking time, and egress time. The access time and egress time are not affected by the train operation plan. In this study, trains are assumed to have the same running time in each section and same dwell time at each station. Hence, different train operation plans make no difference to passengers’ in-train time. This paper focuses on the analysis of passengers’ waiting time cost. Passenger waiting time refers to the time that passengers need to spend at the origin station and transfer station if they are to complete their journey on the line. This is mainly governed by the train route and arrival time through the passenger origin station and transfer station. The calculation of passenger waiting time cost is shown in Eq 3. The objective function of urban rail transit train operation plan optimization problem is shown in Eq 4. The weight coefficient is set to balance the relative importance of operating cost and passengers’ waiting time cost.

$$\begin{array}{c} Z_{2}=c_{3}\sum_{p\in P}[\sum_{f\in F}\sum_{m\in M}(\sum_{r\in R}u_{r,f,m}^{d_{s_{p}^{1},s_{p}^{2}},s_{p}^{1}}-t_{p})\cdot w_{f,m}^{p}] \end{array}$$ (3)

$$\begin{array}{c} \text{min}Z=\tau (Z_{11}+Z_{12})+(1-\tau )Z_{2} \end{array}$$ (4)

### Basic operational rules constraints

To ensure the feasibility and reliability of the train operation plan, Eq 5 indicates that each station on the line is covered by at least one service route. Eq 6 assures that the number of service routes adopted by the line is not greater than the maximum allowable number of service routes operated on the considered line. Eq 7 indicates that the number of train compositions should be smaller than the number of maximum compositions allowed to operate by the service route.

$$\begin{array}{c} \sum_{r\in R}\alpha_{r,s}\cdot x_{r}\geq 1\;\forall s\in S \end{array}$$ (5)

$$\begin{array}{c} \sum_{r\in R}x_{r}\leq \eta \end{array}$$ (6)

$$\begin{array}{c} m\cdot y_{r,f,m}\leq \lambda_{r}\;\forall r\in R,f\in F,m\in M \end{array}$$ (7)

To meet the passenger flow demand and maintain a certain operational service level, Eq 8 indicates that the actual transport capacity of the train operation plan should meet the section passenger flow demand under the expected load *l*. Eq 9 indicates that the number of trains passing through each section of the line should be greater than *a*_*e*_ to ensure the quality of operation service, and less than *b*_*e*_ to ensure that the passing capacity of each section is not exceeded.

$$\begin{array}{c} l\sum_{r\in R}\sum_{f\in F}\sum_{m\in M}o_{m}\cdot \beta_{r,e}\cdot y_{r,f,m}\geq \omega_{e}\;\forall e\in E \end{array}$$ (8)

$$\begin{array}{c} a_{e}\leq \sum_{r\in R}\sum_{f\in F}\sum_{m\in M}\beta_{r,e}\cdot y_{r,f,m}\leq b_{e}\;\forall e\in E \end{array}$$ (9)

On an urban rail transit line, the required number of rolling stocks in the designed operation plan scheme cannot exceed the available fleet size. That is:

$$\begin{array}{c} 2\sum_{r\in R}\sum_{f\in F}y_{r,f,m}-\sum_{r\in R}\sum_{f\in F}\sum_{d\in D}\sum_{r^{\prime }\in R_{r,d}}\sum_{f^{\prime }\in F}\gamma_{r,f,m}^{d,r^{\prime },f^{\prime }}\leq n_{m}\;\forall m\in M \end{array}$$ (10)

### The rolling stock circulation constraints

To ensure the uniqueness of the circulation between the upward and downward trains, Eq 11 is the circulation uniqueness constraint of the train arriving at the turn-back station. Eq 12 is the circulation uniqueness constraint of the train departing from the turn-back station.

$$\begin{array}{c} \sum_{r\in R}\sum_{m\in M}\sum_{r^{\prime }\in R_{r,d}}\sum_{f^{\prime }\in F}\gamma_{r,f,m}^{d,r^{\prime },f^{\prime }}\leq \text{1}\;\forall f\in F,d\in D \end{array}$$ (11)

$$\begin{array}{c} \sum_{r^{\prime }\in R_{r,d}}\sum_{m\in M}\sum_{r^{\prime }\in R_{r,d}}\sum_{f\in F}\gamma_{r,f,m}^{d,r^{\prime },f^{\prime }}\leq \text{1}\;\forall f^{\prime }\in F,d\in D \end{array}$$ (12)

To ensure the reasonability of train circulation at the turn-back station, Eq 13 indicates that the turn-back time of the train at the turn-back station should meet the capacity of turn-back stations, where *M*_1_ is a large enough positive number.

$$\begin{array}{c} \begin{array}{cc} & u_{r^{\prime },f^{\prime },m}^{d^{\prime },s_{r,d^{\prime }}^{1}}-v_{r,f,m}^{d,s_{r,d}^{2}}+M_{1}(1-\gamma_{r,f,m}^{d,r^{\prime },f^{\prime }})\geq \mu_{r,d}\\ & \forall r\in R,f\in F,m\in M,d\in D,r^{\prime }\in R_{r,d},f^{\prime }\in F \end{array} \end{array}$$ (13)

Eq 14 indicates that if the train *f* cannot be circulated, the following trains are also not allowed to be circulated.

$$\begin{array}{c} \begin{array}{cc} & \sum_{r\in R}\sum_{m\in M}\sum_{r^{\prime }\in R_{r,d}}\sum_{f^{\prime }\in F}\gamma_{r,f,m}^{d,r^{\prime },f^{\prime }}\geq \sum_{r\in R}\sum_{m\in M}\sum_{r^{\prime }\in R_{r,d}}\sum_{f^{\prime }\in F}\gamma_{r,f+1,m}^{d,r^{\prime },f^{\prime }}\\ & \forall f\in [1,|F-1|],d\in D \end{array} \end{array}$$ (14)

### Passenger boarding constraints

Eqs 15 and 16 ensure that the departure time of trains available to passengers should be later than the passenger arrival time at origin station, and that the passengers should aboard the train which passes through the passenger’s origin station and destination station. Eq 17 indicates that only one train can be chosen by a passenger.

$$\begin{array}{c} \begin{array}{c} \delta_{r,f,m}^{d,s,t}=\{\begin{array}{cc} 1 & \;\text{if}\;0<t<u_{r,f,m}^{d,s}\\ 0 & \;\text{if}\;u_{r,f,m}^{d,s}\leq t\leq \left|T\right| \end{array}\forall r\in R,f\in F,m\in M,d\in D,s\in S,t\in T \end{array} \end{array}$$ (15)

$$\begin{array}{c} w_{f,m}^{p}\leq \sum_{r\in R}\delta_{r,f,m}^{d,s_{p}^{1},t_{p}}\cdot \alpha_{r,s_{p}^{1}}\cdot \alpha_{r,s_{p}^{2}}\;\forall f\in F,m\in M,d=d_{s_{p}^{1},s_{p}^{2}},p\in P \end{array}$$ (16)

$$\begin{array}{c} \sum_{f\in F}\sum_{m\in M}w_{f,m}^{p}=1\;\forall p\in P \end{array}$$ (17)

Eq 18 is the formula for calculating the number of passengers in the train *f* in direction *d* with composition *m* after leaving station *s*; Eq 19 ensures that the total number of passengers on trains does not exceed train capacity.

$$\begin{array}{c} q_{f,m}^{s,d}=\sum_{p\in P_{s,d}}w_{f,m}^{p}\;\forall s\in S,d\in D,f\in F,m\in M \end{array}$$ (18)

$$\begin{array}{c} q_{f,m}^{s,d}\leq o_{m}\;\forall s\in S,f\in F,m\in M \end{array}$$ (19)

### Variable constraints

The coupling constraints between the variables are as follows: Eq 20 represents the relation between *x*_*r*_ and *y*_*r*,*f*,*m*_. Trains are allowed to run on the service route *r* when the service route *r* is selected. Eq 21 indicates that when the train *f* is not operated, the following trains are also not allowed to be operated. Eq 22 indicates that each train can only be assigned one composition and one route at most.

$$\begin{array}{c} \sum_{f\in F}\sum_{m\in M}y_{r,f,m}\leq |F|\cdot x_{r}\;\forall r\in R \end{array}$$ (20)

$$\begin{array}{c} \sum_{r\in R}\sum_{m\in M}y_{r,f+1,m}\leq \sum_{r\in R}\sum_{m\in M}y_{r,f,m}\;\forall f\in \left[1,|F|-1\right] \end{array}$$ (21)

$$\begin{array}{c} \sum_{r\in R}\sum_{m\in M}y_{r,f,m}\leq 1\;\forall f\in F \end{array}$$ (22)

Eq 23 is the formula of the average train running interval, which is the ratio of the length of the study period to the number of trains.

$$\begin{array}{c} h=\frac{t}{\sum_{r\in R}\sum_{f\in F}\sum_{m\in M}y_{r,f,m}} \end{array}$$ (23)

Eq 24 is the formula for the arrival time of the train *f* in the downward direction at standard station 1. Eq 25 is the formula for the arrival time of the train *f* in the upward direction at the standard station |*S*|.

$$\begin{array}{c} v_{r,f,m}^{d,1}=(f-1)\cdot h\cdot y_{r,f,m}\;\forall r\in R,f\in F,m\in M \end{array}$$ (24)

$$\begin{array}{c} v_{r,f,m}^{d^{\prime },|S|}=[z+(f-1)\cdot h]\cdot y_{r,f,m}\;\forall r\in R,f\in F,m\in M \end{array}$$ (25)

Eq 26 indicates that the arrival time of train *f* in downward at station *s* is equal to the sum of its arrival time at standard station 1 and its travel time from standard station 1 to station *s*. Similarly, according to Eq 27, the arrival time of train *f* in upward at the station *s* can be calculated. Eq 28 indicates that the departure time of the train at the station *s* is equal to the sum of its arrival time and stop time at the station *s*. It is worth noting that the arrival and departure time of train at the station is related to the variable *y*_*r*,*f*,*m*_. When *y*_*r*,*f*,*m*_ = 0, that is, train *f* is not operated, its arrival and departure time at each station is 0.

$$\begin{array}{c} v_{r,f,m}^{1,s}=v_{r,f,m}^{d,1}+t_{1,s}^{d}\cdot y_{r,f,m}\;\forall r\in R,f\in F,m\in M,s\in S\backslash \{1\},d=1 \end{array}$$ (26)

$$\begin{array}{c} v_{r,f,m}^{2,s}=v_{r,f,m}^{d,|S|}+t_{|S|,s}^{d}\cdot y_{r,f,m}\;\forall r\in R,f\in F,m\in M,s\in S\backslash \{|S|\},d=2 \end{array}$$ (27)

$$\begin{array}{c} u_{r,f,m}^{d,s}=v_{r,f,m}^{d,s}+t_{s}^{d}\cdot y_{r,f,m}\;\forall r\in R,f\in F,m\in M,d\in D,s\in S \end{array}$$ (28)

Eqs 29 and 30 indicate that when train *f* and train *f*′ are both operated, the two trains have the possibility of being circulated.

$$\begin{array}{c} \gamma_{r,f,m}^{d,r^{\prime },f^{\prime }}\leq y_{r,f,m}\;\forall r\in R,f\in F,m\in M,d\in D,r^{\prime }\in R,f^{\prime }\in F \end{array}$$ (29)

$$\begin{array}{c} \gamma_{r,f,m}^{d,r^{\prime },f^{\prime }}\leq y_{r^{\prime },f^{\prime },m}\;\forall r\in R,f\in F,m\in M,d\in D,r^{\prime }\in R,f^{\prime }\in F \end{array}$$ (30)

To sum up, the integrated optimization model (IOM) of the train operation planning is shown in Eq 31.

$$\begin{array}{c} \left\{ \begin{array}{c} \text{min}Z\\ \;\text{s.t.}\;(1)∼(3),(5)∼(30) \end{array}\right. \end{array}$$ (31)

## Solution algorithms

### Analysis of model complexity

The integrated optimization model of urban rail transit train operation plan is an integer nonlinear programming model. The decision variables are divided into two categories. One is binary decision variables such as line plan variables and rolling stock schedule variables. The other is positive integer variables such as timetable variables. The number of the decision variables and constraints in the model is determined by the length of the research period |*T*|, the number of stations |*S*|, the number of alternate routes |*R*|, the maximum number of trains in the study period |*F*|, the types of train compositions |*M*|, and the number of passengers |*P*|. The number of variables and constraints in the model is listed in Table 5. Taking the urban rail transit line with 20 stations and 10 alternative routes as an example, the research period is 2 hours, the maximum number of trains is 80, and the train compositions type is 2. The number of variables and constraints in the model is more than 10 million. In addition, the model also contains some nonlinear parts, such as the objective Eqs 3, 15 and 23, etc. Although the model can be transformed into a linear model through linearization, the scale of variables and constraints will be further increased. Considering the feasibility of the solving time of the model, this paper proposes an efficient deterministic search algorithm based on the characteristics of the problem to ensure the optimal solution of the original problem within a reasonable solving time.

**Table 5:** Number of variables and constraints in the model.

Variables or constraints	Max total number
Binary variable *x*_*r*_	|*R*|
Binary variable *y*_*r*,*f*,*m*_	|*R*| × |*F*| × |*M*|
Variable *z*	1
Variable *h*	1
Binary variable $\gamma_{r,f,m}^{d,r^{\prime },f^{\prime }}$	2|*R*| × |*F*|^2^ × |*M*| × |*R*|
Variable $u_{r,f,m}^{d,s}$	2|*R*| × |*F*| × |*M*| × |*S*|
Variable $v_{r,f,m}^{d,s}$	2|*R*| × |*F*| × |*M*| × |*S*|
Variable $q_{f,m}^{s,d}$	2|*F*| × |*M*| × |*S*|
Binary variable $\delta_{r,f,m}^{d,s,t}$	2|*R*| × |*F*| × |*M*| × |*S*| × |*T*|
Binary variable $w_{f,m}^{s,s^{\prime },t}$	|*S*|^2^ × |*F*| × |*M*| × |*T*|
Basic operational rules constraints	4|*S*| + |*R*| × |*F*| × |*M*| + |*M*| − 2
The Rolling Stock Circulation Constraints	6|*F*| + 2|*R*|^2^ × |*F*|^2^ × |*M*| − 2
Passenger boarding constraints	2|*S*| × |*F*| × |*M*| × (|*R*| × |*T*| + 1) + |*F*| × |*M*| × |*P*| + |*P*|
Variable constraints	4|*R*| × |*F*| × |*M*| × (|*S*| + |*R*| × |*F*|) + |*F*|

### Model decomposition

To improve the solving efficiency of the integrated model, the integrated optimization model is decomposed into the line plan optimization sub-model (LPM), timetable and rolling stock schedule optimization sub-model (TRM), and passenger flow allocation sub-model (PAM). The sub-model LPM aims to minimize variable cost *Z*_12_ to optimize the train service routes, train composition, and frequency of each service route during the study period, as shown in Eq 32. Based on the LPM optimization results, TRM optimizes circulation sequence and time of trains to minimize the fixed cost *Z*_11_, as shown in Eq 33. Based on the optimization results of TRM, the sub-model PAM takes minimizing the cost of passenger travel waiting time *Z*_2_ as the goal to determine the variation of passenger flow in trains, as shown in Eq 34. Among them, the sub-model LPM is an integer linear programming model with low complexity, which can be quickly solved to the optimum by commercial solvers such as CPLEX.

$$\begin{array}{c} \left\{ \begin{array}{c} \text{min}Z_{12}\\ \;\text{s.t.}\;(5)∼(9),(20)∼(22) \end{array}\right. \end{array}$$ (32)

$$\begin{array}{c} \left\{ \begin{array}{c} \text{min}Z_{11}\\ \;\text{s.t.}\;(10)∼(14),(23)∼(30) \end{array}\right. \end{array}$$ (33)

$$\begin{array}{c} \left\{ \begin{array}{c} \text{min}Z_{2}\\ \;\text{s.t.}\;(15)∼(19) \end{array}\right. \end{array}$$ (34)

### Deterministic search algorithm

The integrated optimization model of train operation plan aims to minimize the operating enterprise cost and passenger waiting time cost, and the optimal value of the objective function of the original problem is denoted as Z*. When the biggest composition and minimum train interval are adopted for train operation plan during the study period, the enterprise operation cost is the maximum, and the passenger waiting time cost is the minimum. The maximum value of *Z*_12_ is denoted as $Z_{12}^{\text{max}}$. The LPM can be solved by commercial software, the minimum value is denoted as $Z_{12}^{\text{min}}$. Since the LPM can be regarded as a relaxation model of the original problem, the following relation can be obtained, $Z_{12}^{\text{min}}\leq Z_{12}^{*}\leq Z_{12}\leq Z_{12}^{\text{max}}$. Because the range of the LPM can be reasonably estimated and the LPM sub-model is easy to solve, the optimal solution of the original problem can be obtained by traversing the value range of *Z*_12_. Based on the above analysis, the line plan sub-model for iteration (LPMI) is established, as shown in Eq 35. $Z_{12}^{k-1}$ is the optimal value of the objective function in the *k*-1 iteration, and *θ* is the iteration step size. In the iteration process, $Z_{12}^{k}$ always increases at least the difference of travel mileage between the second shortest route and the shortest route in all alternative routes. The value of iteration step is shown in Eq 36, and the calculation formula of maximum iteration times *k*_max_ is shown in Eq 37.

$$\begin{array}{c} \left\{ \begin{array}{c} \text{min}Z_{12}\\ \;\text{s.t.}\;(5)∼(9),(20)∼(22)\\ \;Z_{12}\geq Z_{12}^{k-1}+\theta \end{array}\right. \end{array}$$ (35)

$$\begin{array}{c} \theta =2c_{m}^{2}\cdot \left(g_{2}-g_{1}\right) \end{array}$$ (36)

$$\begin{array}{c} k_{\text{max}}=\frac{\left(Z_{12}^{\text{max}}-Z_{12}^{\text{min}}\right)}{\theta } \end{array}$$ (37)

Based on the decomposition models, a deterministic search algorithm with estimable search range and fixed iteration step size is designed in this study. The parameter symbols in the algorithm are listed in Table 6. The specific process of the deterministic search algorithm is shown in Algorithm 1. Driven by the optimization results of the LPMI, the deterministic search algorithm calls the timetable and rolling stock schedule optimization algorithm shown in Algorithm 2 and the passenger flow allocation algorithm shown in Algorithm 3 in turn. Since these are both deterministic algorithms that meet the constraints of their corresponding sub-models, the optimal solution of IOM can be obtained after finite iterations.

**Table 6:** Parameters in algorithms.

Sets and indices	Definition
*AT*_*s*_	Set of arrival time of trains taking station *s* as destination station;
*DT*_*s*_	Set of departure time of trains taking station *s* as origin station;
*S*_1_	Set of turn-back stations in the urban rail transit line, *S*_1_ ⊂ *S*;
*cN*	Number of rolling stocks circulations;
*dT*_*p*_	Arrival time of passenger *p* at station $s_{p}^{D}$;
*gO*_*p*_	If passenger *p* has left the train, *gO*_*p*_ = 1, 0 otherwise;
*g*_1_	Distance traveled by the train running on the shortest service route in single direction, unit: km;
*g*_2_	Distance traveled by the train running on the second shortest service route in single direction, unit: km;
*oT*_*p*_	Departure time of passenger *p* at station $s_{p}^{O}$;
$pN_{f}^{t}$	The number of passengers on the train *f* at time *t*;
*rT*_*s*_	Minimum turn-back time of the turn-back station *s*, unit: s;
*f*_*p*_	Index of train taken by passenger *p*; -1 indicates that the passenger has not taken any train;
$s_{p}^{o}$	Origin station of passenger *p*;
$s_{p}^{d}$	Destination station of passenger *p*;
*u*	Index of train departure time set;
$u_{f}^{s}$	Departure time of train *f* at station *s*;
*v*	Index of train arrival time set;
Δ*t*	Time increment;

**Algorithm 1 Deterministic search algorithm.**

**Input**: Line basic data; Passenger data;

**Output**: IOM optimal objective function value Z*; Train operation plan;

 **step 1:** Initialize line basic data and iteration step size *θ*. Set Z* = +∞, *k* = 0. The sub-model LPM is solved by a commercial solver.Record the optimal value $Z_{12}^{\text{min}}$. Set $Z_{12}^{k}=Z_{12}^{\text{min}}$. Calculate $Z_{12}^{\text{max}}$ and *k*_max_;

 **step 2:** Determine if *k* = 0, if so, go to Step3. Otherwise, the sub-model LPMI is solved by a commercial solver and record the optimal value $Z_{12}^{k}$;

 **step 3:** Based on $Z_{12}^{k}$, the timetable and rolling stock scheduling optimization algorithm shown in Algorithm 2 is called to solve the sub-model TRM. Record the optimal value of the sub-model $Z_{11}^{k}$. Determine if $Z_{11}^{k}=+\infty$, if so, go to Step5. Otherwise, go to Step4;

 **step 4:** Based on $Z_{12}^{k}$, the passenger allocation algorithm shown in Algorithm 3 is called to solve the sub-model PAM. Record the optimal value of the sub-model $Z_{2}^{k}$. Let $Z^{k}=Z_{11}^{k}+Z_{12}^{k}+Z_{2}^{k}$. Determine if *Z*^*k*^ < Z*, if so, let Z* = *Z*^*k*^;

 **step 5:** Determine if *k* < *k*_max_, if so, go to Step2. Otherwise, end the algorithm.

**Algorithm 2 Timetabling and rolling stock scheduling optimization algorithm.**

**Input**: Set of trains *F*; The value of *k*;

**Output**: TRM optimal objective function value$Z_{11}^{k}$; Timetable; Rolling stock schedule;

 **step 1:** Set the *k*th iteration optimal objective function value $Z_{11}^{k}=+\infty$, temporary objective function value *Z*′_11_ = +∞, *cN* = 0, and offset of the departure time of the first train in the upward direction at the standard station *z* = 0;

 **step 2:** Based on *F* and *z*, the arrival and departure time of the train at the turn-back station is updated. Statistics *DT*_*s*_ and *AT*_*s*_;

 **step 3:** Determine the circulation sequence of trains. The main process is as follows:

  For *s* ∈ *S*_1_ do

   For *v* ∈ *AT*_*s*_ do

    For *u* ∈ *DT*_*s*_ do

     If *v* + *rT*_*s*_ ≤ *u*

     Then *cN* = *cN* + 1, *DT*_*s*_ = *DT*_*s*_/{*u*}, break;

    End

   End

  End

 **step 4:** Set *z* = *z* + 1. Take *cN* as the parameter and update *Z*′_11_ according to the Eq 1. Determine if ${Z^{\prime }}_{11}<Z_{11}^{k}$, if so, set $Z_{11}^{k}={Z^{\prime }}_{11}$;

 **step 5:** Determine if *z* ≤ |*T*|, if so, go to Step2. Otherwise, go to Step6;

 **step 6:** Determine if Eq 10 is satisfied, if not, set $Z_{11}^{k}=+\infty$. End the algorithm.

**Algorithm 3 Passenger allocation algorithm.**

**Input**: Passenger data; The value of *k*;Timetable; Rolling stock schedule;

**Output**: PAM optimal objective function value $Z_{2}^{k}$;

 **step 1:** The passenger flow data set *P* is arranged in ascending order by arrival time *oT*_*p*_. Set *f*_*p*_ = −1 and *gO*_*p*_ = 0 for all passengers. Set the current simulation time *t* = 0;

 **step 2:** Passengers get on the train. The main process is as follows:

  For *p* ∈ *P* do

   If $t\geq dT_{p}\&f_{p}!=-1\&t\geq v_{f_{p}}$

   Then $pN_{f}^{t}=pN_{f}^{t}-1$;

  End

 **step 3:** Passengers get off the train. The main process is as follows:

  For *p* ∈ *P* do

   If *t* ≥ *oT*_*p*_ & *f*_*p*_ = −1

   Then for *f* ∈ *F* do

    If *f* passes by station $s_{p}^{o}\&t<u_{f}^{s_{p}^{0}}\&pN_{f}^{t}<o_{f}$

    Then *f*_*p*_ = *f*, $pN_{f}^{t}=pN_{f}^{t}+1$, break;

   End

  End

 **step 4:** Set *t* = *t* + Δ*t*. Determine if *t* ≤ |*T*|, if so, go to Step2. Otherwise, go to Step5;

 **step 5:** The PAM objective function value $Z_{2}^{k}$ is calculated according to the passenger flow allocation results. End the algorithm;

To prove the optimality of the solution of the deterministic search algorithm, the objective function of IOM in the *k*th iteration is *Z*^*k*^, $Z^{k}=Z_{11}^{k}\text{+}Z_{\text{12}}^{k}\text{+}Z_{\text{2}}^{k}$. The optimal solution of the IOM is Z*, the set of decision variables is *X*, Z* = min*Z*(*X*). The result of the deterministic search algorithm solution is *Z*′. According to the above TPM problem analysis, $Z_{12}^{k}\in \left[Z_{12}^{\text{min}},Z_{12}^{\text{max}}\right]$. When *k* = *i*, $Z_{12}^{i}=Z_{12}^{\text{min}}+\left(i-1\right)\cdot \theta$. Algorithm 2 derives $Z_{11}^{i}=\text{min}Z_{11}^{i}\left(z\right)$ by enumerating the offset of departure time *z*. Algorithm 3 calculates $Z_{2}^{i}$ by enumerating passenger *p*. $Z^{i}=Z_{11}^{i}+Z_{12}^{i}+Z_{2}^{i}$. According to Algorithm 1, *Z*′ = min{*Z*^1^, ⋯, *Z*^*i*^}; Similarly, when *k* = *k*_max_, $Z^{\prime }=\text{min}\left\{Z^{1},\cdots ,Z^{i},\cdots ,Z^{k_{\text{max}}}\right\}$. In essence, the deterministic search algorithm takes the direction of $Z_{12}^{k}$ increasing as the search direction, and discretely enumerates all possible train operation plans through the determined step size *θ*. Hence, $\text{min}\{Z^{1},\cdots ,Z^{i},\cdots ,Z^{k_{\text{max}}}\}$ is equal to min*Z*(*X*). Z* = *Z*′, that is, the proposed algorithm can find the optimal solution of the IOM.

## Numerical experiments

### Case description

**Fig 4:**
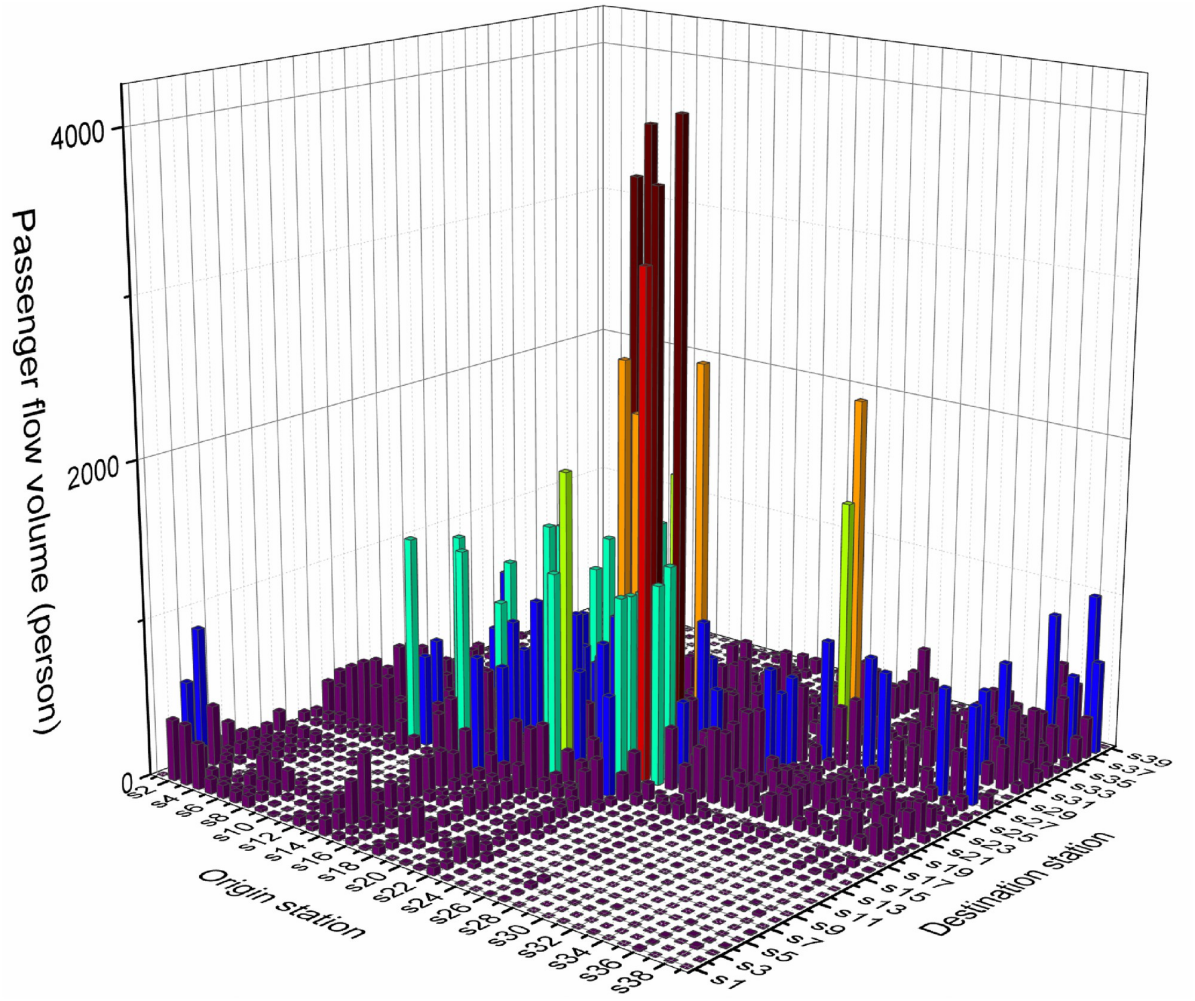
Distribution of line OD passenger flow during the study period.

The experiments reported in this section are based on the operation of the Chongqing Metro Line 3 in China, which is 54 km long, with 39 stations and 38 sections. There are seven optional turn-back stations, among which *s*_1_, *s*_8_, and *s*_14_ only permit the upward arriving train to turn back. Meanwhile, *s*_24_, *s*_28_, *s*_35_ and *s*_39_ only allow the downward arriving train to turn back. The capacity of turn-back stations in each direction is listed in Table 7. It is stipulated that the standard station for downward trains is *s*_1_, and the standard station for upward trains is *s*_39_. There are twelve feasible alternative routes, which are detailed in Table 8. There are currently two types of train compositions operated on the line, namely six-car and eight-car trains. The relevant parameters of train compositions are described in Table 9. The values of other parameters of the model are listed in Table 10. The passenger flow data from 07:00–09:00 during the morning peak period on December 6, 2021 (a working day) is selected to optimize the train operation plan. The distribution of OD passenger flow on the line during the study period is shown in Fig 4. Model optimization results were obtained on a personal computer with Intel Core i7-8550 1.8 GHz CPU and 16 GB memory.

**Table 7:** Minimum turn-back time of turn-back stations.

Station	Downward minimum turn-back time/s	Upward minimum turn-back time /s
*s*_1_	0	300
*s*_8_	0	300
*s*_14_	0	300
*s*_24_	300	0
*s*_28_	300	0
*s*_35_	300	0
*s*_39_	210	0

**Table 8:** Alternate routes’ information.

Train service route	Downward runtime /s	Upward runtime /s	Running kilometrage/km	λ_*r*_
*s*_1_-*s*_24_	3605	3635	31	6
*s*_1_-*s*_28_	4140	4180	35	6
*s*_1_-*s*_35_	5300	5335	46	6
*s*_1_-*s*_39_	6045	6065	53	6
*s*_8_-*s*_24_	2350	2385	18	8
*s*_8_-*s*_28_	2885	2930	22	8
*s*_8_-*s*_35_	4045	4085	33	6
*s*_8_-*s*_39_	4790	4815	40	6
*s*_14_-*s*_24_	1495	1525	12	8
*s*_14_-*s*_28_	2030	2070	16	8
*s*_14_-*s*_35_	3190	3225	27	6
*s*_14_-*s*_39_	3935	3955	34	6

**Table 9:** Rolling stocks’ information.

Train composition	$c_{m}^{1}$ /(CNY/rolling stock)	$c_{m}^{2}$ /[CNY/(rolling stock⋅km)^−1^)]	*n*_*m*_/(rolling stock)	*o*_*m*_/(passengers)
6	340	200	31	962
8	450	250	29	1292

**Table 10:** Other parameters.

Parameter	Value (Unit)
*c*_3_	25 (CNY/h)
*a*_*e*_	6 (trains/h)
*b*_*e*_	24 (trains/h)
*t*	7200 (s)
*l*	100 (%)
*η*	4
*τ*	0.6
Δ*t*	1 (s)

### Outcome analysis

Based on the above data and parameter settings, the C# programming language is used to implement the algorithm. According to the Eq 36, the iteration step *θ* is set to 1200, and after 124 iterations, the deterministic algorithm terminates. The algorithm takes 896.21s in total. Compared with the heuristic algorithm proposed in the relevant literature [5, 6], the deterministic search algorithm can effectively reduce the number of iterations of the search through the determined step size, thus improving the solving efficiency. At the same time, the deterministic algorithm is driven by the linear sub-model LPMI to ensure the optimization of the solution results. The validity of the proposed model is proved by comparing the differences between the model optimization results (MOR) and the manual compilation result (MCR) based on experience in actual operation from the three components of line plan, timetable, and rolling stock schedule.

#### Quality analysis of line planning result

**Fig 5:**
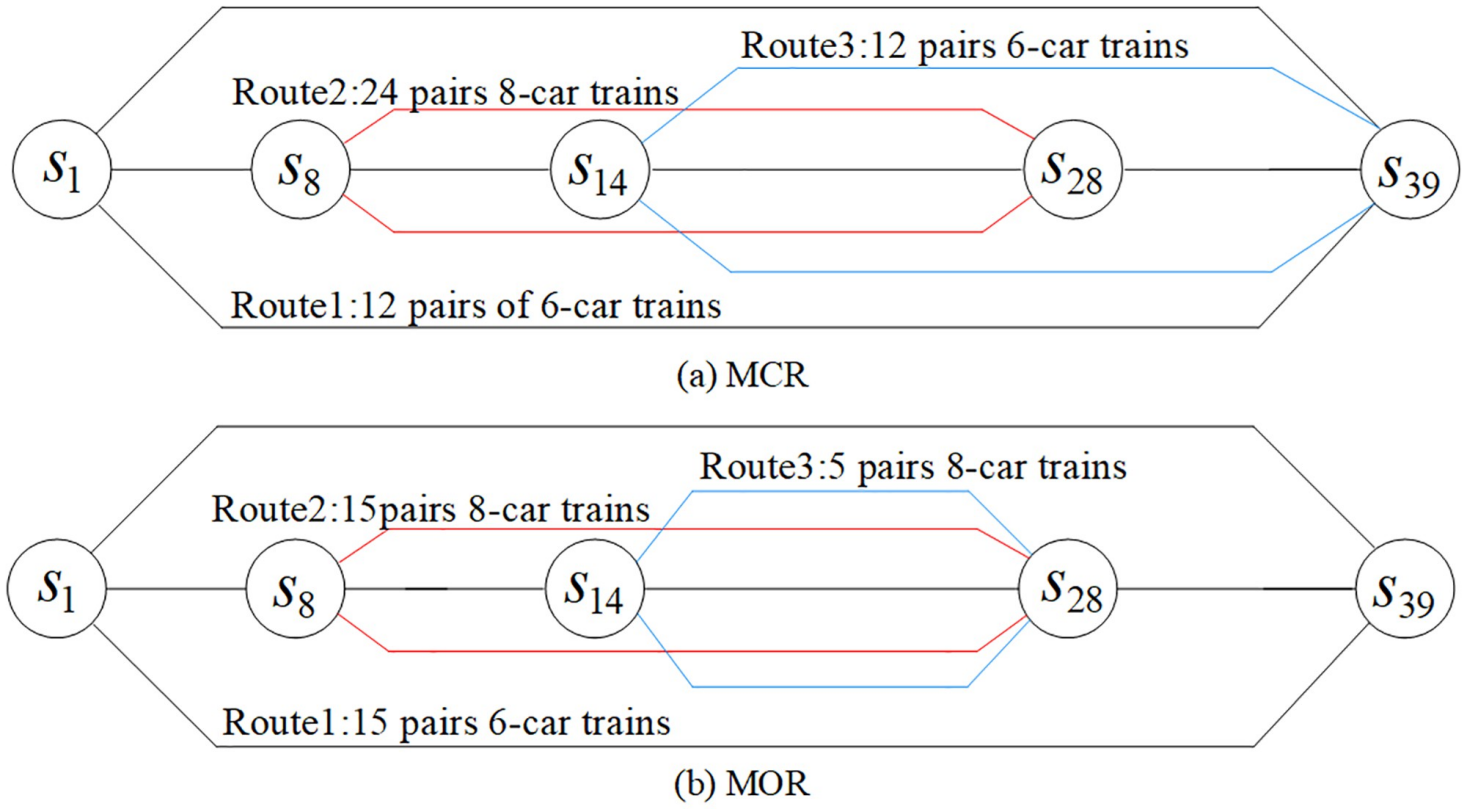
Comparison of line planning results.

**Fig 6:**
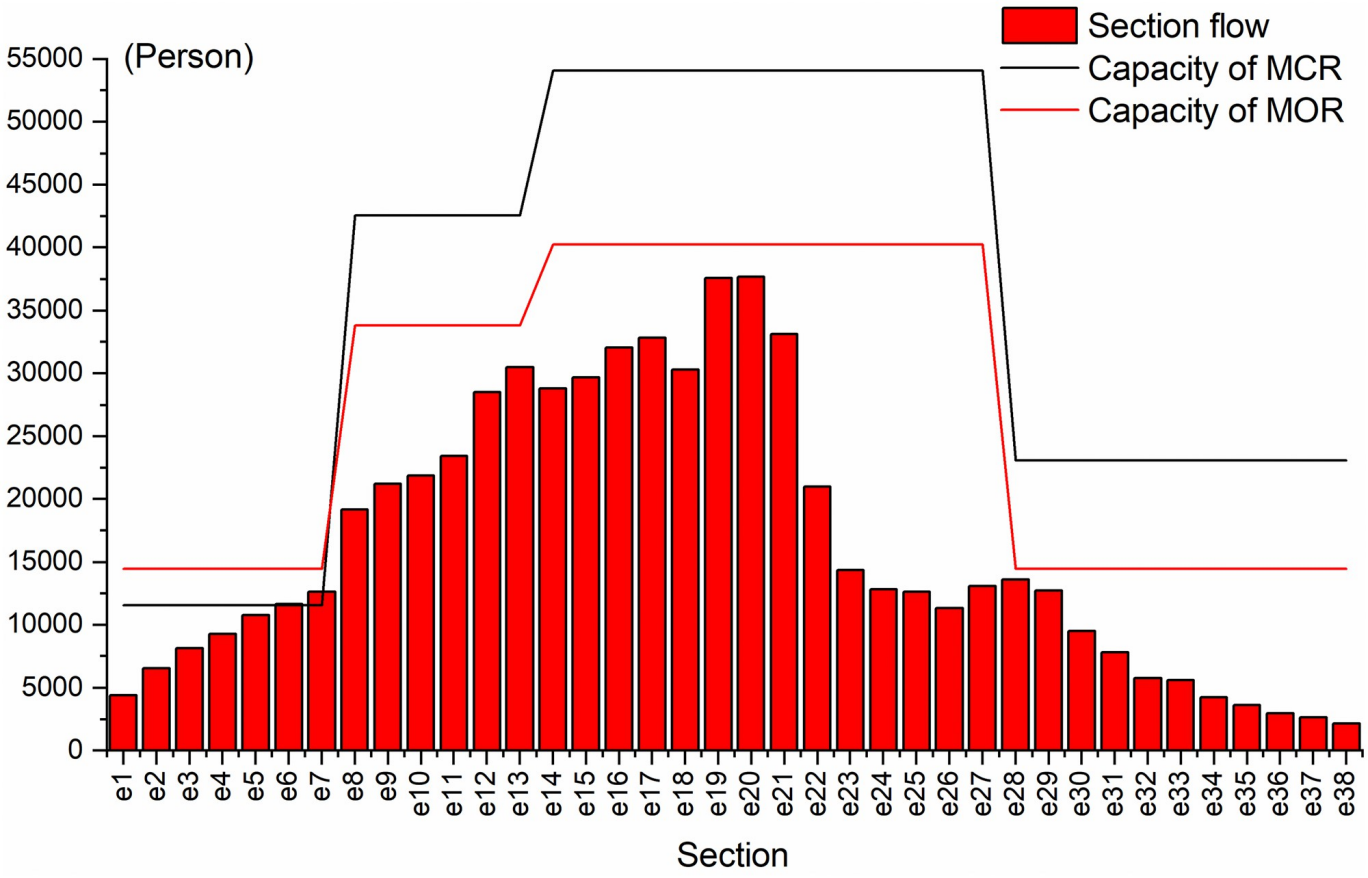
Train capacity and passenger flow demand matching.

**Fig 7:**
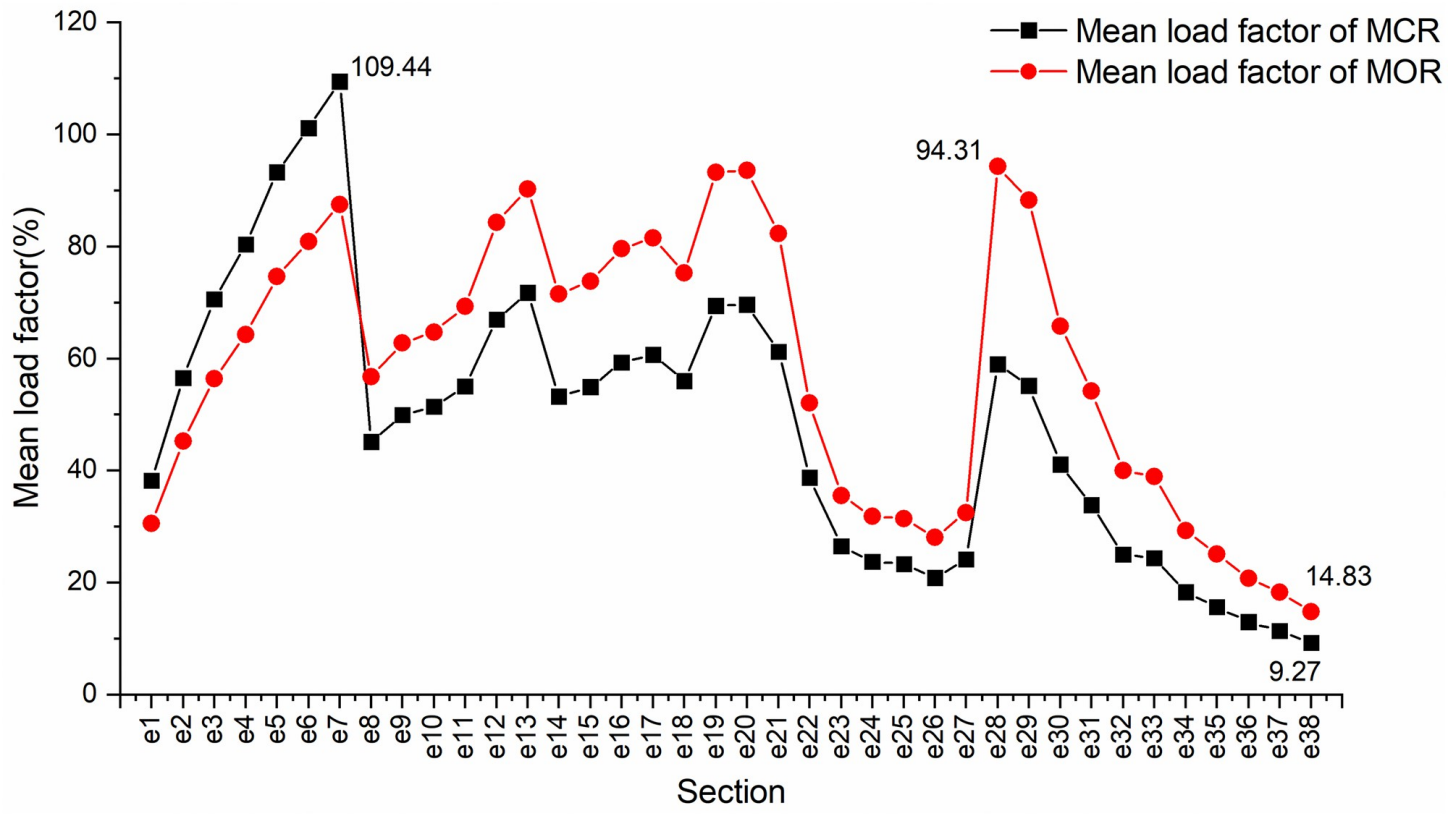
Mean load factor of section.

Fig 5 shows the comparison of line planning results during the study period. The figure indicates the number of pairs of trains on each route within two hours. As shown in Fig 5(a), MCR adopts three service routes of *s*_1_-*s*_39_, *s*_8_-*s*_28_, and *s*_14_-*s*_39_, among which the *s*_8_-*s*_28_ operates eight-car trains. The ratio of trains running on the service routes is 1:2:1, with an average running interval of 150 s. In contrast, as shown in Fig 5(b), MOR adopts three service routes of *s*_1_-*s*_39_, *s*_8_-*s*_28_, and *s*_14_-*s*_28_, among which the *s*_8_-*s*_28_ and *s*_14_-*s*_28_ operate eight-car trains. The average running interval of the trains is 205 s. The comparison of transport capacity and passenger demand matching is shown in Fig 6. The MCR cannot meet the passenger demand of section *e*_7_, and there is a waste of transport capacity from section *e*_8_ to *e*_28_. MOR solves the problem of tight transport capacity in section *e*_7_ by increasing the number of trains running on the service route *s*_1_-*s*_39_. At the same time, MOR selects the service route *s*_14_-*s*_28_, and fully reduces the waste of transport capacity from section *e*_8_ to *e*_28_ by adopting the operation mode of large-composition trains and long-running intervals. The mean section load factor during the study period is shown in Fig 7, of which the maximum section load factor during the MCR period is 109.44%, and the minimum section load factor is 9.27%. The MOR has a maximum section load factor of 94.31% and a minimum section load factor of 14.83%, which has a better load factor stability than that of MCR.

#### Timetabling and rolling stock scheduling quality analysis

**Fig 8:**
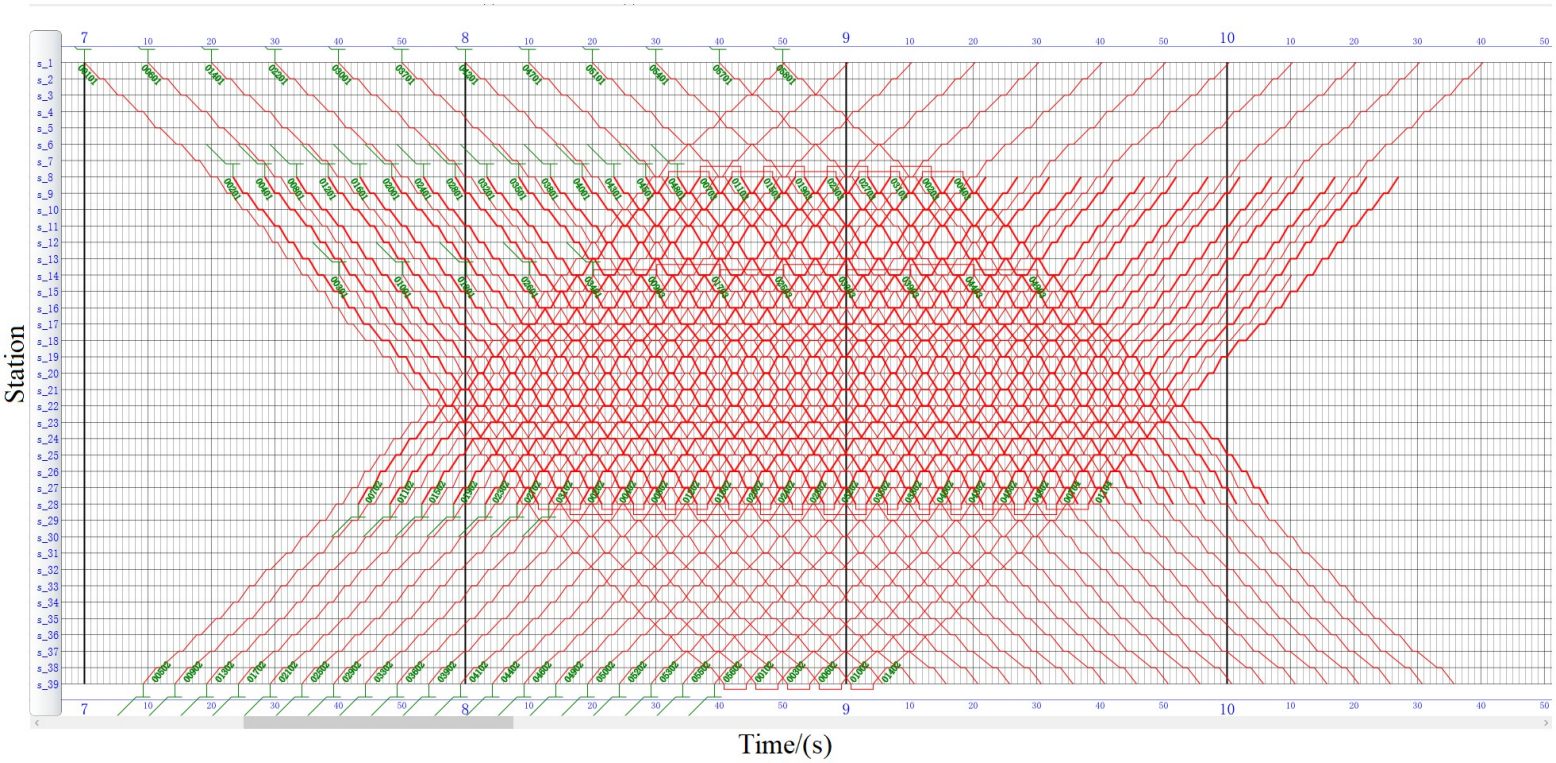
MCR’s train diagram.

**Fig 9:**
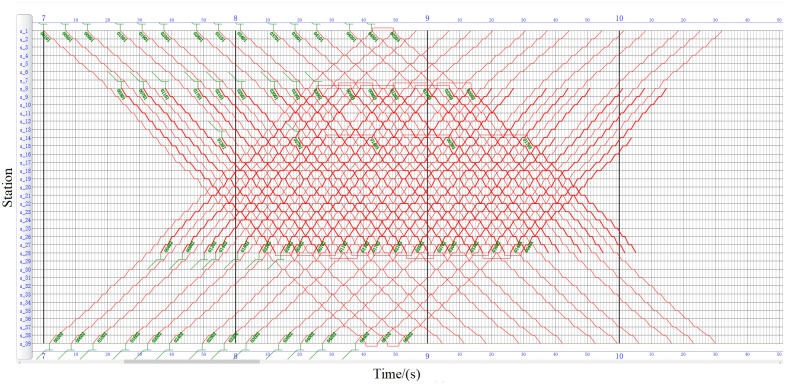
MOR’s train diagram.

As shown in Figs 8 and 9, the timetable and the train rolling stock schedule in the study period are compared and displayed in the form of train diagram. The first upward train offset of MCR is 555 s, and the first upward train offset of MOR is 105 s according to calculation. Affected by the long line mileage, in the MCR, the upward trains ending at station *s*_1_ cannot be circulated within the period, making the entry and exit depots of subsequent trains more complicated. In addition, the running time of MOR’s is generally higher than that of MCR at the turn-back station. In the actual operation process, under the condition of not increasing the fleet size and meeting the constraints of the turn-back station, the longer the running time at the turn-back station, the more time is left for the train turn-back running and passengers to get on and off the train. This makes the MOR train operation plan more robust than MCR. The fleet size required for the turn-back station can be counted by the number of trains arriving and departing at the turn-back station and the number of circulations. The detailed comparison of train circulation at the turn-back station is listed in Table 11. The fleet size required by MOR is thirteen lower than that of MCR, delivering fixed cost reduction and efficiency improvements while meeting the demand for passenger flow.

**Table 11:** MOR’s (MCR’s) rolling stock schedule of turn-back stations.

Turn-back station	Number of departure trains	Number of circulations	Fleet size	Fleet size gap
*s*_39_	15(12)	1(0)	14(12)	2
*s*_28_	15(24)	6(9)	9(15)	-6
*s*_14_	5(12)	3(7)	2(5)	-3
*s*_8_	20(24)	13(17)	7(7)	0
*s*_1_	15(24)	2(5)	13(19)	-6
Total	70(96)	25(38)	45(58)	-13

#### Model optimization quality analysis

The detailed comparison of the objective function values of each part is shown in Table 12. Due to the large number of trains in MCR and the small interval between trains, the average waiting time for passengers is 117s. Compared with MCR, the passenger waiting time of MOR is 145s, which is 28s longer. However, MOR’s enterprise fixed cost and variable cost decrease more, and the comprehensive objective function expense decreases by 13%. In addition, MOR can not only meet the demand for passenger flow, but also greatly reduce the number of rolling stocks in use. The saved rolling stocks can be used to perform special transportation tasks such as express trains and cross-line trains, so as to improve the passenger service level of the network, but this part of the benefit can not be directly reflected in the comparison of the objective function value. To sum up, the optimization method proposed in this paper can carry out integrated optimization across the line planning, timetabling, and rolling stock scheduling considering the facilities and equipment conditions of urban rail transit lines and the characteristics of passenger flow, and in doing so it can deliver better outcomes than those of the empirical method in actual production.

**Table 12:** Objective function value statistics.

Objective function	MOR	MCR	Variation
Enterprise fixed cost/CNY	10368.0	13614.0	-24%
Enterprise variable cost /CNY	156742.4	204603.1	-23%
Passenger waiting time cost/CNY	71773.8	58111.6	24%
Average waiting time of passengers/s	145	117	28s
Total cost /CNY	238884.2	276328.7	-13%

### Sensitivity analysis

#### Fleet size

To explore the influence of the fleet size on the design of the train operation plan, based on the rolling stock of the optimal train operation plan, MOR, the number of six-car rolling stock and eight-car rolling stock were increased or decreased respectively, as shown in Table 13. Five groups of optimized train operation plans were solved and observed under different fleet sizes. Train operation plans with serial numbers 1 and 2 revealed that although the plan utilized a smaller fleet size than the original problem, they could still support the delivery of the train operation plan. Compared with train operation plan 2, train operation plan 3 increases the number of eight-car rolling stock and reduces the number of six-car rolling stock. The optimized train operation plan increases the number of eight-car rolling stock and reduces the six-car rolling stock. Contrary to train operation plan 3, train operation plan 4 reduces the number of eight-car rolling stock and increases the number of six-car rolling stock. The optimized train operation plan reduces the number of eight-car rolling stock and increases the number of six-car rolling stock. Train operation plans 3 and 4 show that when any train composition’s fleet size in the train operation plan is lower than the demand for the fleet size in the optimal train operation plan, the model will adjust the optimal result to adapt to the current fleet size. Train operation plan 5 shows that when the fleet size used in the train operation plan cannot meet the basic passenger flow demand, there is no feasible train operation plan.

**Table 13:** Fleet size sensitivity analysis.

No	*n*_6_/trains	*n*_8_/trains	Line planning result	*z*_1_/CNY	*z*_2_/CNY	*z*/CNY
1	35	35	*s*_1_-*s*_39_:15 pairs of six-car trains;	167110.4	71773.8	238884.2
			*s*_8_-*s*_28_:15 pairs of eight-car trains;			
			*s*_14_-*s*_28_:5 pairs of eight-car trains.			
2	29	16	*s*_1_-*s*_39_:15 pairs of six-car trains;	167110.4	71773.8	238884.2
			*s*_8_-*s*_28_:15 pairs of eight-car trains;			
			*s*_14_-*s*_28_:5 pairs of eight-car trains.			
3	25	23	*s*_1_-*s*_39_:12 pairs of six-car trains;	165449.3	78605.6	244054.9
			*s*_8_-*s*_28_:24 pairs of eight-car trains.			
4	32	15	*s*_1_-*s*_39_:13 pairs of six-car trains;	167500.2	75246.0	242746.2
			*s*_8_-*s*_28_:13 pairs of eight-car trains;			
			*s*_14_-*s*_28_:13 pairs of six-car trains.			
5	10	10	-	-	-	-

#### The maximum allowable number of routes

The maximum number of routes allowed to operate on the line is an important parameter that affects the train operation plan. In this paper, the value of *η* is set from 1 to 5, and the corresponding model is solved. The calculation results are described in Table 14. As can be seen from Table 14, while the optimization results of the model are the same when *η* ≥ 3, the total cost gradually rises as the maximum allowable number of routes operated on the line is reduced. When *η* becomes smaller, the travel interval at some stations becomes smaller too. Although the passenger service level is improved, the operating cost of enterprises will increase even more, which will lead to an increase in the total cost. When *η* is 1, that is, when the line adopts the full-length service route, the model cannot find the feasible boundary due to insufficient fleet size.

**Table 14:** Maximum allowable number of routes analysis.

No	*η*	Line planning result	*z*_1_/ CNY	*z*_2_/CNY	*z*/CNY
1	5	*s*_1_-*s*_39_:15 pairs of six-car trains;	167110.4	71773.8	238884.2
		*s*_8_-*s*_28_:15 pairs of eight-car trains;			
		*s*_14_-*s*_28_:5 pairs of eight-car trains.			
2	4	*s*_1_-*s*_39_: 15 pairs of six-car trains;	167110.4	71773.8	238884.2
		*s*_8_-*s*_28_:15 pairs of eight-car trains;			
		*s*_14_-*s*_28_:5 pairs of eight-car trains.			
3	3	*s*_1_-*s*_39_: 15 pairs of six-car trains;	167110.4	71773.8	238884.2
		*s*_8_-*s*_28_:15 pairs of eight-car trains;			
		*s*_14_-*s*_28_: 5 pairs of eight-car trains.			
4	2	*s*_1_-*s*_39_: 14 pairs of six-car trains;	194219.2	67104.1	261323.3
		*s*_8_-*s*_28_: eight pairs of six-car trains;			
		*s*_8_-*s*_28_:16pairs of eight-car trains.			
5	1	-	-	-	-

#### Weight coefficient

**Fig 10:**
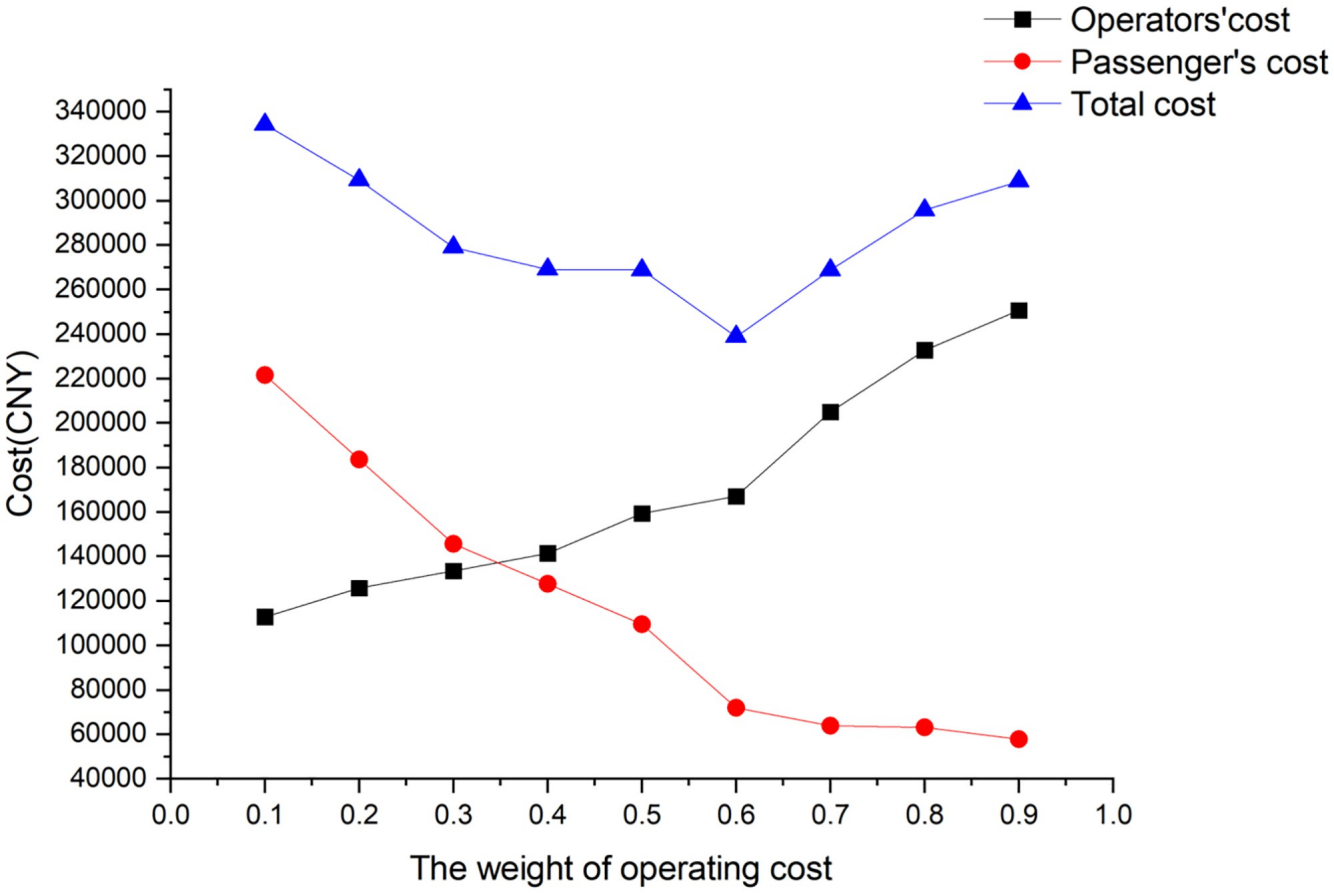
Adjustment results of weight factors.

The setting of the weight coefficient *τ* can effectively balance the cost of enterprise operation and passenger waiting time. In this research, the value of *τ* is set from 0.1 to 0.9, and each model is solved. The solution results are shown in Fig 10. The operating cost of the enterprise and the cost of passenger waiting time conflict with each other. When the value of *τ* is equal to 0.1, the enterprise operating cost reaches the minimum value, and the passenger travel cost reaches the maximum value. With the increase of *τ*, the cost of the enterprise operation gradually increases, while the cost of passengers’ waiting time gradually decreases, reaching their maximum and minimum values, when the value of *τ* is equal to 0.9. Influenced by the preparation of the train operation plan in the actual operation of the enterprise, this paper sets *τ* to 0.6.

## Conclusion

This research takes passenger flow demand and line fixed facilities and equipment conditions as input, and studies the integrated optimization problem of urban rail transit train operation plans. An integer nonlinear programming model was established to minimize the cost of enterprise operation and passenger waiting time. The model was decomposed and a deterministic algorithm was designed to realize the collaborative optimization of line planning, timetabling, and rolling stock scheduling. Computational analysis shows that this integrated optimization model can better reduce the operating cost of enterprises compared with the train operation plan prepared by stages based on manual experience. The fixed cost can be reduced by 24%, the variable cost can be reduced by 23%, and the total cost can be reduced by 13%. Although passenger waiting time increases in the integrated optimization model, it can deliver a more reasonable train capacity allocation according to the distribution characteristics of the line passenger flow. The maximum section load factor decreased from 109.44% to 94.31%, and the minimum section load factor increased from 9.27% to 14.83%, which improves the passenger service level of the train operation plan. At the same time, the model comprehensively considered the line planning, timetable, and rolling stock schedule. Therefore, it can accurately calculate the fleet size, making the model optimization results easier for operators to implement. It is worth noting that, although the model does not explicitly involve constraints on the proportion of trains running between routes, the resulting optimized train operation plan tends to be periodic to ensure the successful circulation between trains within the period as much as possible, as well as to reduce the fleet size and the running kilometrage of trains. This periodicity is in line with the actual production demand and it also serves to make the optimized train operation plan more reasonable.

This paper mainly studies the integration of urban rail transit operation plans during peak hours when passenger flow fluctuation is small. In the next step, it is necessary to explore the integration of train operation plans for the large fluctuation of passenger flow during the transition period between off-peak hours and peak hours.
